# Noggin Over-Expressing Mouse Embryonic Fibroblasts and MS5 Stromal Cells Enhance Directed Differentiation of Dopaminergic Neurons from Human Embryonic Stem Cells

**DOI:** 10.1371/journal.pone.0138460

**Published:** 2015-09-18

**Authors:** Mi-Sun Lim, Min-Seop Shin, Soo Young Lee, Yang-Ki Minn, Jeong-Kyu Hoh, Youl-Hee Cho, Dong-Wook Kim, Sang-Hun Lee, Chun-Hyung Kim, Chang-Hwan Park

**Affiliations:** 1 Graduate School of Biomedical Science and Engineering, Hanyang University, Seoul, Korea; 2 Hanyang Biomedical Research Institute, Hanyang University, Seoul, Korea; 3 Department of Microbiology, College of Medicine, Hanyang University, Seoul, Korea; 4 Department of Obstetrics and Gynecology, College of Medicine, Hanyang University, Seoul, Korea; 5 Department of Medical Genetics, College of Medicine, Hanyang University, Seoul, Korea; 6 Department of Biochemistry and Molecular Biology, College of Medicine, Hanyang University, Seoul, Korea; 7 Department of Neurology, Hangang Sacred Heart Hospital, Hallym University, Seoul, Korea; 8 Department of Physiology and Cell Therapy Center, Yonsei University College of Medicine, Seoul, Korea; 9 Institute of Green Bio Science and Technology, Seoul National University, Pyeongchang, Korea; University of Texas at Austin Dell Medical School, UNITED STATES

## Abstract

Directed methods for differentiating human embryonic stem cells (hESCs) into dopaminergic (DA) precursor cells using stromal cells co-culture systems are already well established. However, not all of the hESCs differentiate into DA precursors using these methods. HSF6, H1, H7, and H9 cells differentiate well into DA precursors, but CHA13 and CHA15 cells hardly differentiate. To overcome this problem, we modified the differentiation system to include a co-culturing step that exposes the cells to noggin early in the differentiation process. This was done using γ-irradiated noggin-overexpressing CF1-mouse embryonic fibroblasts (MEF-noggin) and MS5 stromal cells (MS5-noggin and MS5-sonic hedgehog). After directed differentiation, RT-PCR analyses revealed that engrailed-1 (*En-1*), *Lmx1b*, and *Nurr1*, which are midbrain DA markers, were expressed regardless of differentiation stage. Moreover, tyrosine hydroxylase (*Th*) and an A9 midbrain-specific DA marker (*Girk2*) were expressed during differentiation, whereas levels of *Oct3/4*, an undifferentiated marker, decreased. Immunocytochemical analyses revealed that protein levels of the neuronal markers TH and TuJ1 increased during the final differentiation stage. These results demonstrate that early noggin exposure may play a specific role in the directed differentiation of DA cells from human embryonic stem cells.

## Introduction

Neurodegenerative disorders including Parkinson’s disease (PD), Alzheimer’s disease, and Huntington’s disease are characterized by progressive neural loss and dysfunction [1]. Currently available treatments for neurodegenerative disorders are only symptomatic and can’t prevent disease progression, necessitating an efficient therapy like cell replacement therapy. Various cell sources have been used for cell transplantation, for example fetal and adult neural precursor cells (NPCs). However, these cells have a low proliferation rate and are therefore not ideal for transplantation [2]. Human embryonic stem cells (hESCs)-derived and human-induced pluripotent stem cells (hiPSC)-derived NPCs/dopamine (DA) neurons could potentially provide an unlimited source for cell transplantation [3–6]. Although several differentiation methods have been shown to generate tyrosine hydroxylase (TH)-positive neurons from hESCs and hiPSCs in vitro, the differentiation efficiency was low, and the transplanted cells were unable to survive in the host brain [7–10]. To improve the directed differentiation of hESCs and hiPSCs to appropriate NPCs/DA neurons, several studies have used the BMP signaling antagonist noggin to promote neural differentiation from the ectoderm [11] through suppressed SMAD signaling [12–14] and rostral induction. Noggin is expressed in the nervous system and has been verified to be an essential factor for neural induction [15]. In this study, we investigated directed differentiation of H9, HSF6, CHA13, and CHA15 hESCs into NPCs/DA neurons. Expression of phosphorylated SMAD (pSMAD) of CHA hESCs (CHA13 and CHA15) was higher than that of H9 and HSF6 hESCs. We hypothesized that the high expression level of pSMAD was responsible for the lower survival and differentiation of CHA hESCs than H9 and HSF6 hESCs. To test this hypothesis, we exposed hESCs to noggin early in the differentiation process by co-culturing CHA13 and CHA15 hESCs with noggin-overexpressing feeder/stromal cells. As a result early noggin exposure markedly reduced pSMAD protein expression and induced rosette-like structure formation by CHA hESCs.

## Materials and Methods

### hESC culture and in vitro differentiation

**Fig 1:**
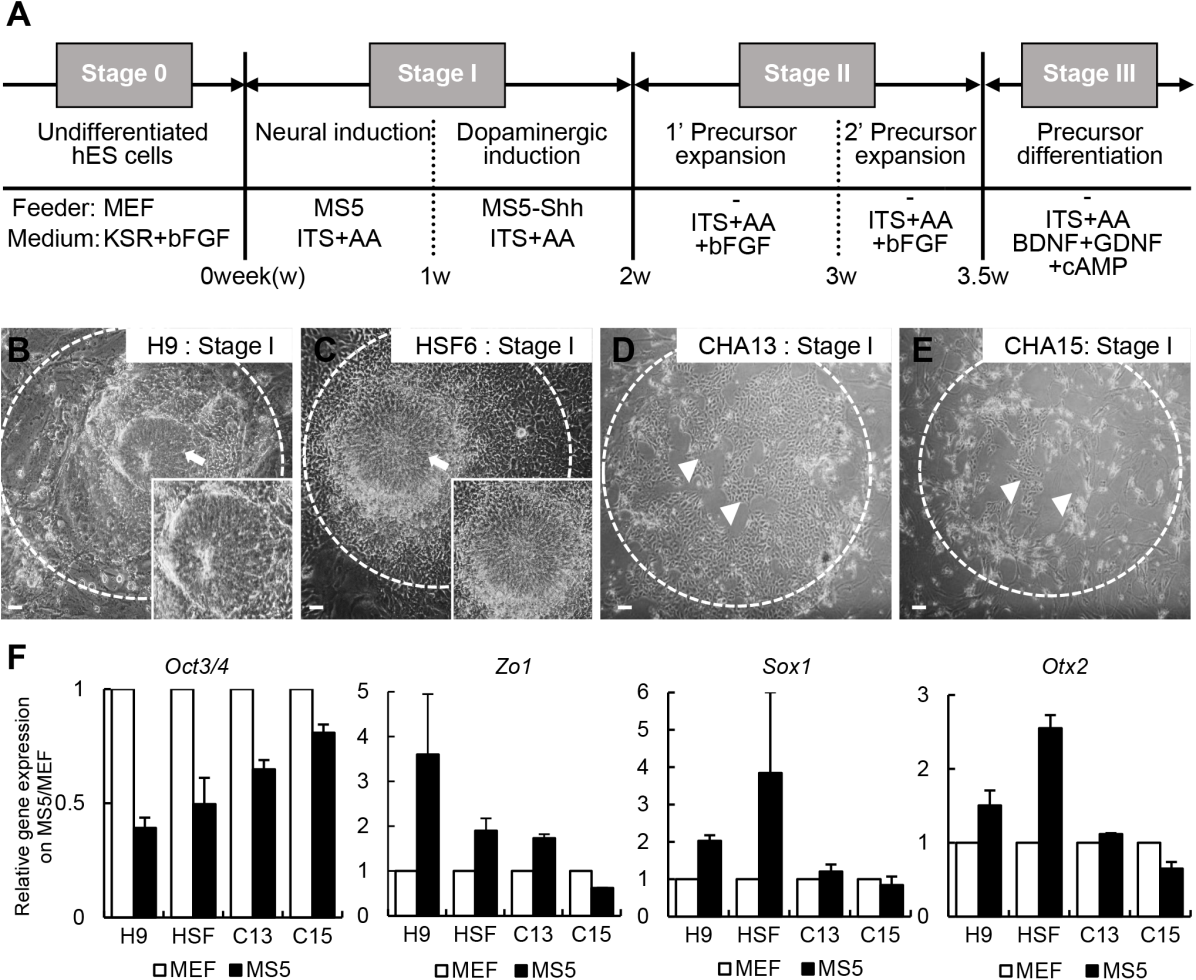
Low efficiency induction of neural rosette-like cells from the hESCs CHA13 and CHA15 using a previously published stromal cell co-culture method. (A) A protocol to generate NPCs/DAs from hESCs is depicted. (B-E) Morphology of H9 (B), HSF6 (C), CAH13 (D), and CHA15 (E) cells under neural induction (stage I) conditions (on γ-irradiated MS5 feeder layer in ITS+AA medium). H9 and HSF6 cells formed neural rosette structures with a neuroepithelial cell morphology (arrows in B and C). However, CHA13 and CHA15 cells showed only limited production of rosette structures under stage I conditions (arrowheads in D and E; note that most CHA13 and 15 cells were detached from the plate). (F) Gene expression analysis. CHA hESCs did not express neuronal markers (*Zo1*, *Sox1* and *Otx2*) nor did they show decreased levels of the pluripotent marker *Oct3/4* compared to H9 and HSF6 hESCs. Scale bar = 100 μm.

The hESC culture protocol (HYE-08-03) was approved by Hanyang University Institutional Review Board. Undifferentiated hESCs, namely CHA13, CHA15 (established at CHA Stem Cell Institute, Seoul, Korea), HSF-6 (established at University of California, San Francisco, CA, USA), and H9 (WiCell, Madison, WI, USA) were maintained as described previously [7]. Briefly, undifferentiated hESCs were grown on a feeder layer of γ-irradiated mouse embryonic fibroblasts (MEFs) (CF1, Charles River Kingston, Kingston, NY, USA) in hESC medium [DMEM/F12 (Invitrogen, Grand Island, NY, USA) supplemented with 20% (v/v) serum replacement (Invitrogen), 0.1 mM nonessential amino acids (Invitrogen), 0.1 mM mercaptoethanol (Sigma-Aldrich, St. Louis, MO, USA), and 4 ng/mL basic fibroblast growth factor (bFGF, R&D Systems, Minneapolis, MN, USA)]. Medium was changed daily. To induce neural differentiation, we used two co-culture methods with stromal cells. In the first co-culture system, hESC colonies were cultured onto a feeder layer of γ-irradiated MEFs for 1 week before transferring the colonies to a feeder layer of γ-irradiated MS5 stromal cells (kindly provided by Dr. Kwang-Soo Kim, Harvard University) for 7–10 days, after which they were passaged onto γ-irradiated MS5 cells stably expressing sonic hedgehog (MS5-shh) for an additional 7–10 days in medium containing insulin/transferrin/selenium with 0.2 mM ascorbic acid (Sigma, ITS+AA) [16] (Fig 1A). In the second co-culture system, hESC colonies were cultured onto a feeder layer of γ-irradiated MEFs stably expressing noggin (MEF-noggin) for 1 week in hESC medium (undifferentiation medium); these cells were then passaged onto γ-irradiated MS5 cells stably expressing noggin (MS5-noggin) for an additional 7–10 days. Finally, the cells were cultured on γ-irradiated MS5-shh for 7–10 days in ITS+AA [17]. At the end of the co-culturing step, the hESC colonies had differentiated to form neural structures i.e. hESC-NPC colonies or rosettes. Rosette-forming cells were harvested from stromal feeders, and gently triturated by pipetting the cells into small cluster-sized cells in ITS+AA supplemented with 20 ng/mL bFGF, followed by re-seeding on poly _L_-ornithine (PLO, 15 μg/mL, Sigma)/fibronectin (FN, 1 μg/mL, Sigma)-coated culture plates. After culturing for 7 days in ITS+AA+bFGF to expand the NPCs, rosette-forming cells were dissociated into single cells by incubation in Ca^2+^/Mg^2+^-free Hank’s balanced salt solution (HBSS, Invitrogen) for 1 hour. Cells were replated on culture dishes for maintenance in ITS+AA+bFGF medium and/or transferred to PLO/FN coated glass coverslips for phenotypic determination in the absence of bFGF and presence of 20 ng/mL brain-derived neurotrophic factor (BDNF, R&D Systems), 20 ng/mL glia cell line-derived neurotrophic factor (GDNF, R&D Systems), and 0.5 mM dibutyryl cAMP (Sigma).

### Retroviral production

A retroviral plasmid for noggin expression was constructed by engineering the noggin DNA fragment (GI:1710364) into the retroviral vector IRES3-EGFPBsd-CL [18]. The retroviral vector was transfected into 293GPG packaging cells using Lipofectamine 2000 reagent (Invitrogen). Supernatants containing viral particles were harvested 72 hours after transfection.

### Reverse transcriptase-polymerase chain reaction (RT-PCR)

Total cellular RNA was isolated using TRI REAGENT (Molecular Research Center, Inc. Cincinnati, OH, USA), and cDNA was synthesized from 5 μg of total RNA in a 20 μl reaction volume using the Superscript kit (Invitrogen) according to the manufacturer’s instructions. PCR conditions are provided in S1 Table.

### Immunostaining of cultured cells

Immunostaining of cultured cells was performed as described previously [19]. Cells were photographed using epifluorescence and confocal microscopy (Leica Microsystems, Wetzlar, Germany). Primary antibody information is summarized in S2 Table.

### Cytosolic and nuclear fractionation

To prepare nuclear extracts, cells were washed with cold phosphate-buffered saline (PBS). Cells were then harvested in microcentrifuge tubes and centrifuged at 300 × g for 4 min at 4°C. The supernatants were discarded, and the pellets were resuspended in 400 μl of cold buffer A [10 mM HEPES (pH 7.9), 10 mM KCl, 0.1 mM EDTA, 1 mM DTT, 0.5 mM phenylmethylsulfonyl fluoride (PMSF, Sigma)] and incubated on ice for 15 min. Next, 25 μl of 10% Nonidet P-40 (NP40, Sigma) was added, and the mixtures were vortexed briefly. Nuclei were pelleted by centrifugation at 2800 × g for 4 min at 4°C and then resuspended in 50 μl of ice-cold buffer B [20 mM HEPES (pH 7.9), 0.4 M NaCl, 1 mM EDTA, 1 mM DTT, 1 mM PMSF]. Mixtures were shaken vigorously for 15 min at 4°C, centrifuged at 15,000 × g for 5 min, and the supernatants were collected as the cytosolic fraction.

### Western blot analysis

To determine protein levels, we prepared protein extracts from undifferentiated hESCs. Undifferentiated hESCs were isolated from feeder cells by mechanical methods. Cells were lysed by incubation with radio-immunoprecipitation assay (RIPA) buffer (50 mM Tris-HCl, 150 mM NaCl, 1% sodium deoxycholate, 1% NP40, 0.1% SDS, pH 7.4) containing 1 mM PMSF and protease inhibitor cocktail (Roche, IN, USA) on ice. Suspended cells in lysis buffer were sonicated on ice and centrifuged at 15,000 × G for 20 minutes at 4°C. Proteins were quantified using Bradford reagent (BIO-RAD, Hercules CA, USA), and 50 μg samples of extracted protein were resolved on SDS-polyacrylamide gels and transferred to nitrocellulose membranes. Membranes were incubated with primary antibodies at 4°C overnight and then incubated with secondary antibody coupled to horseradish peroxidase. Immunoreactivity was visualized using enhanced chemiluminescence (WelProt^TM^HRP detection kit, WelGENE, Daegu, Korea). Protein bands were quantified with a densitometer (Molecular Devices, VERSAmax, CA, USA).

### Cell counting and statistical analyses

Cell counting was performed with uniform random selection of 5–10 microscopic fields/well with 3–4 wells per experimental condition. All values were confirmed with at least three independent experiments. Data are expressed as means ± SEM. When more than two groups were compared, a paired *t*-test was performed using SigmaPlot for Windows, version 10.0, (Systat Software GmbH, Erkrath, Germany).

## Results

### The MS5 stromal cell co-culture system does not induce significant neural differentiation of CHA13 and CHA15 hESCs

Protocols for DA induction of hESCs based on co-culture with MS5 or PA6 stromal cells have been reported [8, 16, 20, 21]. Four hESCs, namely H9, HSF6, CHA13, and CHA15, were differentiated by a co-culturing method (Fig 1A). Briefly, each hESCs (stage 0) was seeded on a γ-irradiated MS5 feeder layer (stage I, neural induction) for 7–10 days in ITS+AA medium. H9 and HSF6 cells differentiated into neural rosette-like cells, which are primitive neuroepithelial cells (Fig 1B and 1C, insets are high magnification views). However, CHA13 and CHA15 cells did not form an adequate amount of rosette structures (Fig 1D and 1E), and cell survival was poor, thus progression to stages II-III could not continue. In addition, gene expression patterns of CHA hESCs were different from those of H9 and HSF6 hESCs. After differentiation on the MS5 feeder layer, CHA hESCs still expressed high levels of the pluripotent marker, *Oct3/4*, whereas rosette (*ZO1*) and neural ectoderm markers (*Sox1 and Otx2*) were expressed at much lower levels than in H9 and HSF6 hESCs (Fig 1F). These results indicated that not all of the hESCs differentiated into DA precursor cells using the classical stromal cell co-culture system.

### Both phosphorylated SMAD1/5/8 and phosphorylated SMAD2/3 are highly expressed in CHA hESCs compared to H9 and HSF6 cells

**Fig 2:**
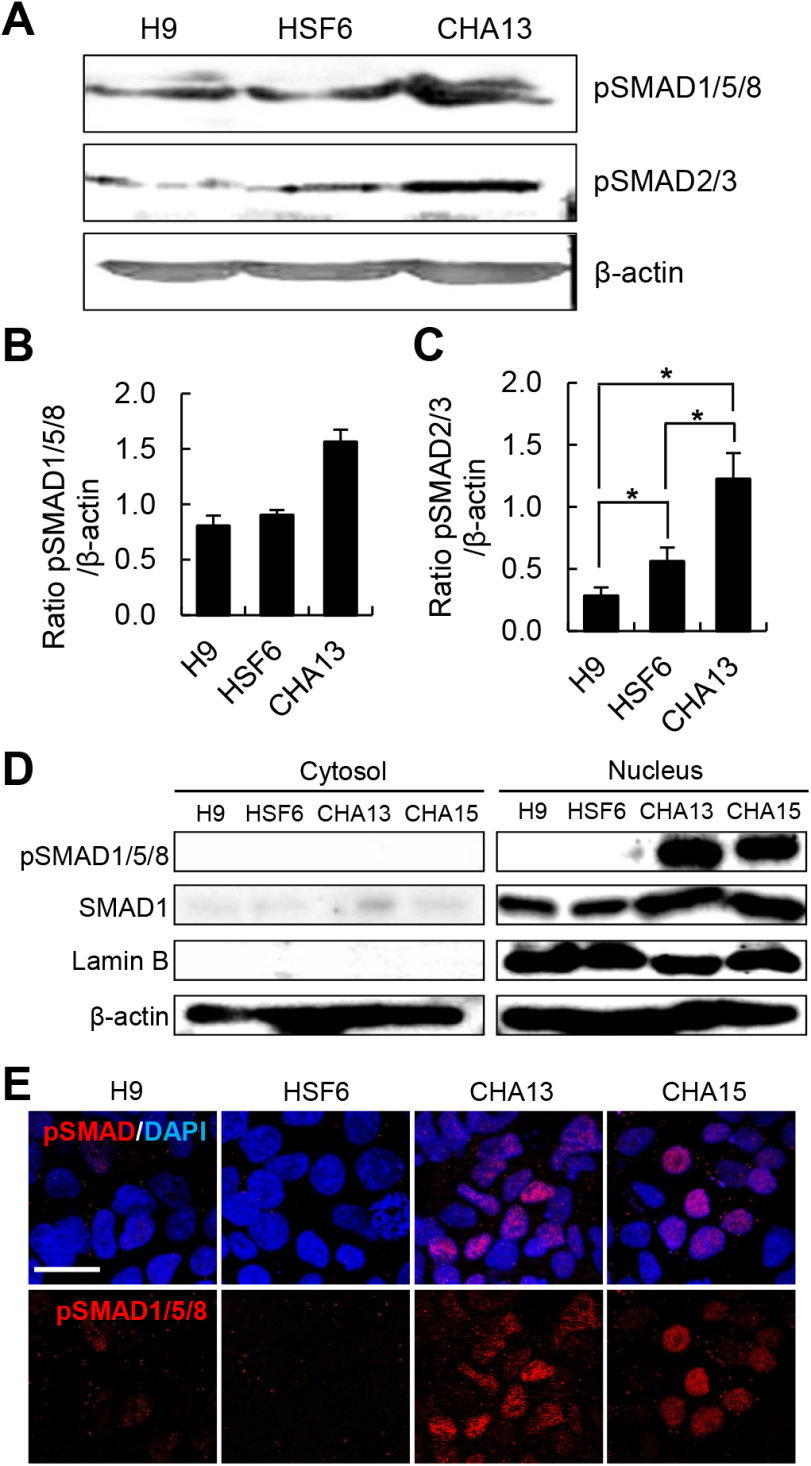
pSMAD protein expression is upregulated in CHA hESCs. (A-C) pSMAD protein expression was analyzed by Western blot. The BMP pathway related proteins, pSMAD1/5/8 and pSMAD2/3, were highly expressed in undifferentiated CHA13 hESCs compared to H9 and HSF6 hESCs. (D) Pattern of pSMAD1/5/8 expression in H9, HSF6, CHA13, and CHA15 hESCs after cytosolic/nuclear fractionation. Undifferentiated CHA 13/15 hESCs show high levels of pSMAD in the nucleus. SMAD1 expression was used as a proxy of total SMAD protein levels. Lamin B was used to confirm equal loading of nuclear proteins, and β-actin was used as a loading control. (E) Confocal microscopic image. CHA 13/15 cells expressed high levels of pSMAD1/5/8 (red) in the nucleus. However, pSMAD expression was lower in the nuclei of H9 and HSF6 cells or absent. All data are means ± S.E. of three independent experiments *p<0.05 compared with H9 and HSF6 cells. Scale bar = 20 μm.

**Fig 3:**
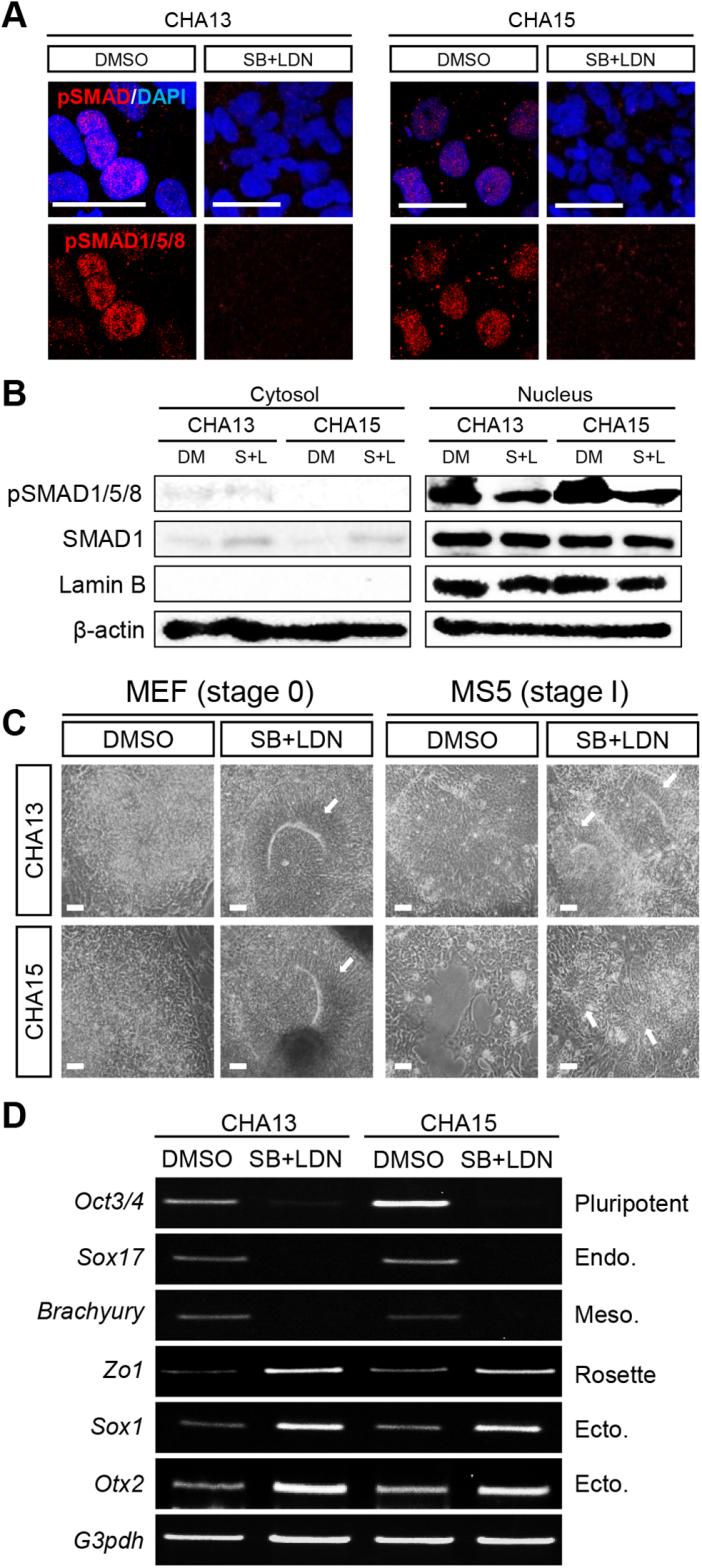
Efficient neural conversion of CHA13/15 hESCs by dual SMAD signaling inhibition. (A) Two SMAD inhibitors, SB431542 (SB) and LDN193189 (LDN)-treated cells did not express pSMAD in the nucleus based on confocal image analysis. (B) SB+LDN-treated cells showed decreased pSMAD expression in the nucleus by Western blot analysis. SMAD1 was used to assess the total SMAD protein level, Lamin B was used to confirm equal loading of nuclear proteins, and β-actin was used as a loading control. (C) SB and LDN were added to CHA13 and 15 hESCs grown on a MEF-feeder layer stage (stage 0). After 7 days, neuroepithelial-like structure were observed (white arrows) in the SMAD inhibitor-treated group (SB+LDN) compared to the control group (DMSO). After stage 0, the MS5-feeder layer stage (stage 1) were consistently treated with both SMAD inhibitors. At the end of this stage, rosette structures appeared more under SB+LDN culture conditions (white arrows) than the DMSO culture condition. (D) Gene expression analysis. SB+LDN treated cells lost expression of the pluripotent marker, *Oct3/4*, the endoderm marker *Sox17*, and the mesoderm marker *Brachyury*. In contrast, expression of rosette (*Zo1*) and ectoderm (*Sox1* and *Otx2*) markers increased. Scale bar = 20 μm (A) 100 μm (C).

To determine why CHA13 and 15 cells failed to form rosette structures, we focused on the BMP signaling related proteins, SMAD1/5/8 and SMAD2/3, which are known to inhibit neural induction. We evaluated expression of phosphorylated SMAD1/5/8 (pSMAD1/5/8) and phosphorylated SMAD2/3 (pSMAD2/3) proteins in undifferentiated H9, HSF6, and CHA13 hESCs. Interestingly, CHA13 cells expressed the highest levels of pSMAD1/5/8 and pSMAD2/3 (Fig 2A–2C), indicating that the failure of CHA13 cells to differentiate to DA neurons was likely due to the higher expression of pSMAD in these hESCs than the other hESCs. To confirm increased pSMAD activity, we analyzed pSMAD protein localization by western blot and immunofluorescence after cell fractionation. H9 and HSF6 hESCs showed low or no pSMAD expression in the nucleus whereas CHA 13/15 hESCs expressed high level of pSMAD1/5/8 in the nucleus (Fig 2D and 2E). These supported our hypothesis that activation of pSMAD in the nucleus led to inhibition of the neuronal differentiation of hESCs. Several studies have demonstrated that treatment of hESCs and hiPSCs with two SMAD inhibitors, SB431542 (SB) and LDN193189 (LDN), enhanced neural generation [12–14]. Based on this, we performed neural induction using a dual-SMAD-inhibition protocol. As expected, expression of pSMAD1/5/8 was decreased (Fig 3A and 3B) and undifferentiated CHA13/15 cells adopted neural rosette-like structures (Fig 3C) in the SB+LDN treated condition. In addition, SB+LDN treatment led to ectodermal induction (*Zo1*, *Sox1*, and *Otx2*) but blocked endodermal (*Sox17*) and mesodermal (*Brachyury*) lineage specification (Fig 3D).

### In vitro differentiation of CHA13 and CHA15 hESCs to NPC/DA neurons by early exposure to the BMP antagonist, noggin

**Fig 4:**
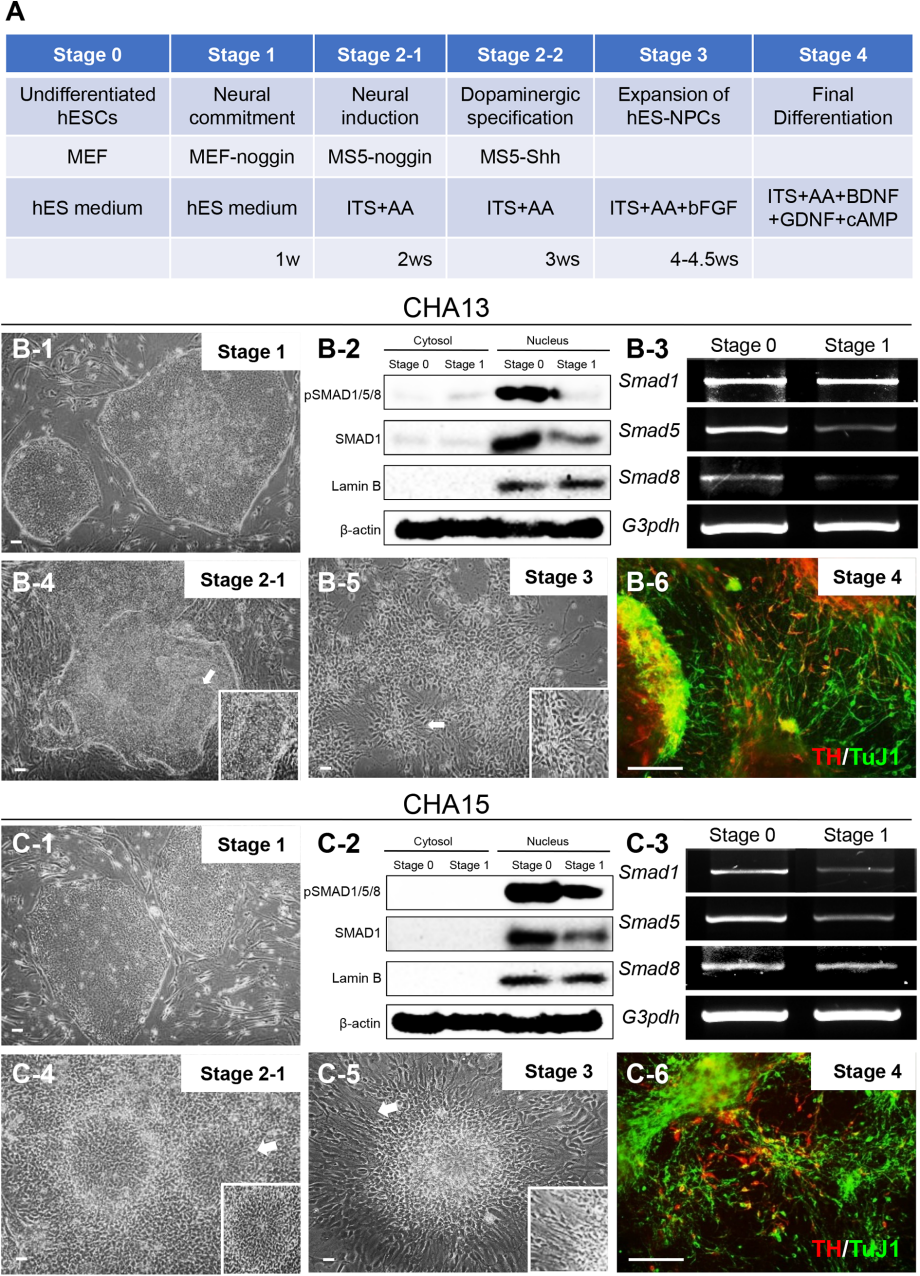
Early exposure of noggin can generate rosette-like cells as well as DA neuronal precursor cells from CHA13 and 15 hESCs. (A) The protocol to generate NPCs/DAs from CHA13 and 15 hESCs using early exposure of noggin is illustrated schematically. In our study, hESCs were exposed to noggin at stage 1 by co-culturing noggin over-expressing MEF cells (MEF-noggin) in hESC medium. CHA cells showed an altered morphology during the neural commitment period (7–10 days) on MEF-noggin cells (B-1 and C-1: stage 1), and pSMAD1/5/8 and *Smad1*, *5*, *8* expression decreased during the period of noggin exposure (B-2 and C-2; western blot, B-3 and C-3; RT-PCR, respectively). The neural induction period for 7–10 days on MS5-noggin stromal cells (B-4 and C-4; stage 2–1, inset; early rosette structure-arrows), dopaminergic specification during 7–10 days on MS5-shh stromal cells in ITS + AA medium. Rosette structured cells were mechanically isolated and attached to FN-coated dishes in ITS + AA + bFGF medium to expand hESC-NPCs (B-5 and C-5; stage 3, inset; NPC morphology-arrow). NPCs were incubated for 10 days in the absence of bFGF and in the presence of BDNF, GDNF and cAMP for final differentiation. Representative image of TH/TuJ1-positive neurons derived from CHA hESCs (B-6 and C-6; stage 4). Scale bar = 100 μm.

**Fig 5:**
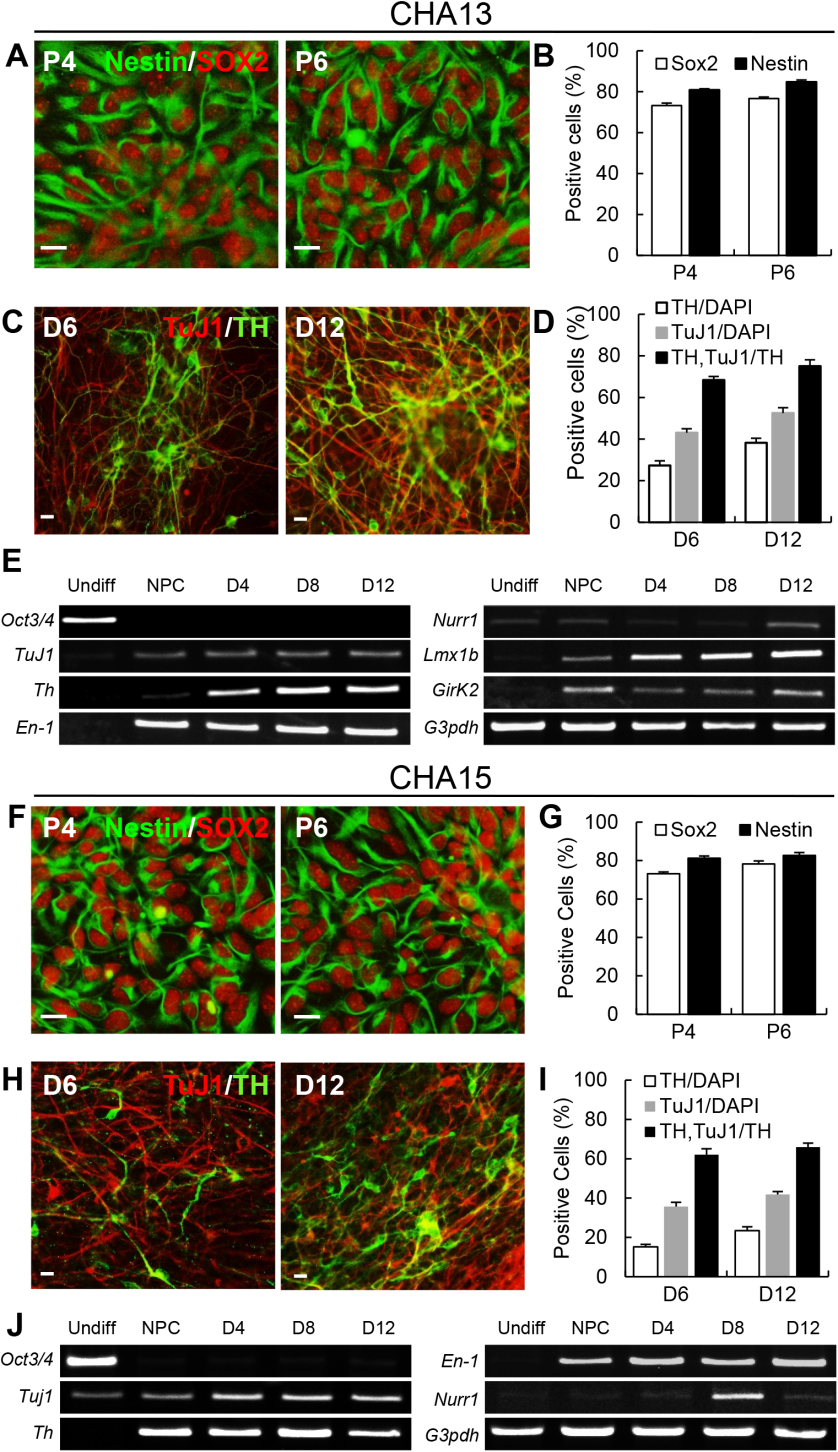
Characterization of CHA13/15-NPCs derived using the noggin overexpressing method. After dissociation of rosette-forming cells, their NPC characteristics were investigated. (A-B) CHA13-NPCs were stably expandable without loss of self-renewing potential. NPCs were cultured for more than six passages and analyzed by immunocytochemistry for the NPC markers nestin (green) and SOX2 (red), and the proliferating marker ki67 (data not shown). (C-D) The proportion of TH/TuJ1-positive cells increased during the final differentiation period: D6, differentiation for 6 days; and D12, differentiation for 12 days. (E) Expression of genes specific for midbrain development during in vitro differentiation. Midbrain development-related genes, *Th*, *En-1*, *Nurr1*, *Lmx1b*, and *Girk2*, as well as the neuronal related gene *TuJ1* were expressed. Conversely, expression of the pluripotent marker *Oct3/4* decreased during differentiation. *G3pdh* is the control housekeeping gene. (F-J) Characterization of CHA15-NPCs. CHA15-NPCs also showed NPC properties (F-G) and DA neuron generation (H-J). Undiff, undifferentiated hESCs; NPCs, neural precursor cells; D4, D6, and D12, differentiation for 4, 6, and 12 days, respectively. Scale bar = 20 μm.

Given that high pSMAD protein expression blocked the formation of neural rosette structures, we hypothesized that neural differentiation could potentially be enhanced by treatment of the cells with noggin, which is a pSMAD suppressor (14). We cultured CHA13/15 hESCs on noggin-expressing cells during the early stage of co-culture differentiation (Fig 4A). Undifferentiated CHA13 and CHA15 cells were transferred onto γ-irradiated MEF-noggin feeder cells for 7–10 days before placing them on a MS5-noggin feeder layer. At the end of the stage 1 process, a change in the morphology of the hESCs was evident (Fig 4B-1 and 4C-1, stage 1). Moreover, pSMAD1/5/8 protein and *Smad* 1, 5, 8 mRNA levels decreased during stage 1 (Fig 4B-2, 3 and 4C-2, 3) compared to levels of these markers in hESCs grown on MEF feeder cells. After 7–10 days, stage 1 cells were split into small clusters, re-seeded on γ-irradiated MS5-noggin cells for 7–10 days (stage 2–1), and then transferred to γ-irradiated MS5-shh cells for another 7–10 days (stage 2–2). Previous studies have established that shh is a crucial factor in the specification of midbrain DA neurons for mouse ES cell differentiation in culture [22]. Under our culture conditions, rosette structures were clearly observed as shown in Fig 4B-4 and 4C-4 (Insets are high magnification views). Next, we isolated the rosette–like cells mechanically and seeded them on a PLO/FN coated culture dish under ITS + AA + bFGF culture conditions [Fig 4B-5 and 4C-5, stage 3, hESC-derived neural precursor cells (hES-NPCs)]. hES-NPCs were continuously expanded following passages. After the final differentiation step, cells expressed the neuronal marker TuJ1 and DA marker TH by immunofluorescence (Fig 4B-6 and 4C-6, stage 4). CHA13-derived NPCs expressed the NSC-specific markers nestin and SOX2 (Fig 5A and 5B). These cells were stably expandable without loss of self-renewing potential (Fig 5A and 5B, P4; SOX2, 73.2 ± 1.24%, nestin, 80.8 ± 0.58%, P6; SOX2, 76.6 ± 0.72%, nestin, 84.7 ±1.03%). During the final differentiation step, the proportion of TuJ1+ cells (TuJ1/DAPI) and TH+ cells (TH/DAPI) increased as well as the proportion of TH+ cells out of TuJ1+ cells (TH,TuJ1/TH) [Fig 5C and 5D, D6; 27.3 ± 2.22% (TH/DAPI), 43.0 ± 1.94% (TuJ1/DAPI), 68.3 ± 1.71% (TH,TuJ1/TH), D12; 38.2 ± 2.15% (TH/DAPI), 52.5 ± 2.56% (TuJ1/DAPI), 75.0 ± 3.02% (TH,TuJ1/TH)] based on immunostaining. Semi-quantitative RT-PCR analyses revealed that expression of markers of midbrain DA development, including *En-1*, *Nurr1* and *Lmx1b*, was induced during in vitro differentiation (Fig 5E). Expression of the A9-type (nigral) mDA neuron marker *Girk2* was also detected (Fig 5E). Similarly, CHA15-derived NPCs also effectively generated NPC and DA neurons (Fig 5F–5J). However, *Lmx1b* and *Girk2* were expressed at low levels in CHA15-derived DA neurons. These findings suggested that highly expressed pSMAD signaling was mitigated by early inhibition of pSMAD signaling by noggin, resulting in the generation of NPCs/DA neurons.

## Discussion

The main goal of this study is to develop a strategy to enhance the induction of hESC-derived NPC/DA neurons in vitro using a stromal cell co-culturing method. Although several strategies have already been published, the differentiation efficiencies and experimental protocols are highly diverse. To improve efficiency, SMAD signaling has been targeted using the combination of noggin and SB431542 as a differentiation strategy [23–26]. The addition of noggin elevated the differentiation rates of neural lineages of hESCs [23] and hiPSCs [27]. We found that CHA13 and 15 hESCs did not form rosette-structure cells unlike H9 and HSF6 hESCs when cultured using a published stromal cell co-culture method [7]. We found that pSMAD protein expression levels vary among hESCs. We hypothesized that the low differentiation efficiency was related to the level of pSMAD expression. We overexpressed noggin in feeder/stromal cells at an early stage (MEF-noggin and MS5-noggin) using a co-culture method. Interestingly, pSMAD1/5/8 and pSMAD2/3 protein expression decreased during differentiation stage 1 culture conditions compared to the undifferentiated stage (stage 0). By applying our method, CHA hESCs can be differentiated into neural rosette forming cells and NPCs. Procedures to establish CHA13 and 15-derived NPCs through exposure to MEF-noggin, MS5-noggin, and MS5-shh resulted in maintenance of neural precursor properties. Our results are consistent with those of a previous study that demonstrated that hESC-derived NPCs are a suitable and stable source of cells for transplantation [28]. Parkinson’s disease is one of the most common neurodegenerative diseases and is characterized by selective and progressive loss of DA neurons in the substantia nigra pars compacta [29]. hESC-derived midbrain-like DA neurons are a potential cell source for cell transplantation. We also demonstrated that the CHA13 and 15 hESC-derived DA neurons expressed TuJ1 and were TH-positive during the differentiation period. Expression of the midbrain specific genes, *En-1*, *Nurr1*, *Lmx1b*, and *Girk2*, was observed during differentiation, whereas levels of the pluripotent marker, *Oct3/4*, decreased significantly. These results are consistent with those of a previous study [25], which suggested that SMAD inhibition during hESC differentiation resulted in loss of OCT3/4 expression. To investigate whether noggin overexpression enhances DA neuron differentiation of H9 hESCs, we generated H9 cell-derived NPCs/DA neurons using the noggin overexpression feeder method. Unexpectedly, TH+ and TuJ1+ cell populations were not significantly increased relative to control cells. We guess that this is because SMAD expression is already low in undifferentiated H9 hESCs (S1 Fig). Our noggin over-expressing feeder/stromal cell co-culture system is simple and can be easily applied to the neural differentiation of hESCs and hiPSCs. It also has the additional benefit of cost savings, because using the noggin overexpressing feeder/stromal cell is far less expensive than using noggin cytokine. Despite such advantages, this method does require a longer culture period for rosette formation and generation of NPCs. This may be because noggin overexpressing feeders are less effective than cytokine treatment at decreasing pSMAD protein levels. Several studies have reported that simultaneous application of two SMAD inhibitors, noggin and SB431542, has a synergistic effect on neural differentiation. SB431542, an inhibitor of TGFβ signaling, inhibits SMAD signaling through suppression of activin/ALK4 signaling [30, 31]. Based on these previous studies together with our findings, we intend to add SB431542 to our co-culture system based on the noggin over-expressing feeder/stromal cells in future studies.

In conclusion, we developed the first noggin-overexpressing feeder/stromal cells co-culture method to stimulate hESCs to develop into NPCs/DA neurons. Investigation of CHA13 and 15 hESCs showed that robust expression of pSMAD is a barrier for neural induction. In this study, we differentiated hESCs using a noggin over-expressing feeder/stromal cells. This noggin-overexpressing feeder/stromal cells co-culture method can be used to convert hESCs into neural and DA neuron lineages. Taken together, the early application of noggin overcome non-neural induction of hESCs and promoted DA precursor establishment.

## Supporting Information

S1 Table Gene-specific primer sequences and RT-PCR conditions. (DOCX)

S2 Table Primary antibody information. (DOCX)

S1 Fig Noggin does not enhance the dopaminergic differentiation of H9 hESCs. (DOCX)
