# Supplementary material for: Noggin Over-Expressing Mouse Embryonic Fibroblasts and MS5 Stromal Cells Enhance Directed Differentiation of Dopaminergic Neurons from Human Embryonic Stem Cells
Source: PLoS One. 2015 Sep 18;10(9):e0138460. doi: 10.1371/journal.pone.0138460 (PMC4575120; doi:10.1371/journal.pone.0138460)
Supplement: S1 Table — (DOCX) [file pone.0138460.s001.docx]

**Supplemental Table 1: Gene-specific primer sequences and RT-PCR conditions.**

| Gene | PCR Sequence  (5’-3’) | Annealing  Temperature | Cycle | | Product Size |
| --- | --- | --- | --- | --- | --- |
| Oct3/4 | F: CTTGCTGCAGAAGTGGGTGGAGGAA | 55℃ | | 35 | 168bp |
|  | R: CTGCAGTGTGGGTTTCGGGCA |  |  |  |  |
| TuJ1 | F: CAACAGCACGGCCATCCAGG | 58℃ | | 35 | 244bp |
|  | R: CTTGGGGCCCTGGGCCTCCGA |  |  |  |  |
| TH | F: GAGTACACCGCCGAGGAGATTG | 62℃ | | 35 | 279bp |
|  | R: GCGGATATACTGGGTGCACTGG-3 |  |  |  |  |
| En1 | F: GCAACCCGGCTATCCTACTTATG | 60℃ | | 35 | 247bp |
|  | R: ATGTAGCGGTTTGCCTGGAAC |  |  |  |  |
| Pitx3 | F: GGAATCGCTACCCTGACATGAG | 60℃ | | 35 | 276bp |
|  | R: TGAAGGCGAACGGGAAGGTCT |  |  |  |  |
| Nurr1 | F: TTCTCCTTTAAGCAATCGCCC | 60℃ | | 35 | 332bp |
|  | R: AAGCCTTTGCAGCCCTCACAG |  |  |  |  |
| Lmx1b | F: ACGAGGAGTGTTTGCAGTGCG | 60℃ | | 29 | 253bp |
|  | R: CCCTCCTTGAGCACGAATTCG |  |  |  |  |
| GirK2 | F: GCTACCGGGTCATCACAGAT | 60℃ | | 35 | 162bp |
|  | R: ACTGCATGGGTGGAAAAGAC |  |  |  |  |
| SMAD1 | F: GAGACCGCTTTATTTCACCATATC C | 60℃ | | 30 | 881bp |
|  | R: CATAGTAGACAATAGAGCACCAGTGTTTT |  |  |  |  |
| SMAD5 | F: CGGTAGCCACTGACTTTGAGTTAC | 60℃ | | 30 | 377bp |
|  | R: AGCTGAAATGGACTTCCTGGTC |  |  |  |  |
| SMAD8 | F: TGC TGT GGC CTC TTA TGC AC | 60℃ | | 30 | 420bp |
|  | R: GTC TCCACCCGGCGG |  |  |  |  |
| G3PDH | F: GCTCAGACACCATGGGGAAGGT | 55℃ | | 35 | 474bp |
|  | R: GTGGTGCAGGAGGCATTGCTGA |  |  |  |  |
