# Supplementary material for: Noggin Over-Expressing Mouse Embryonic Fibroblasts and MS5 Stromal Cells Enhance Directed Differentiation of Dopaminergic Neurons from Human Embryonic Stem Cells
Source: PLoS One. 2015 Sep 18;10(9):e0138460. doi: 10.1371/journal.pone.0138460 (PMC4575120; doi:10.1371/journal.pone.0138460)
Supplement: S2 Table — (DOCX) [file pone.0138460.s002.docx]

**Supplemental Table 2: Primary antibody information.**

| Antibody | Dilution | | Company | Location | |
| --- | --- | --- | --- | --- | --- |
| Polyclonal antibody (Rabbit) | | | | | |
| Nestin #130 | 1:50 | Provide by Dr. R. McKay | | | NIH, Bethesda, MD, USA |
| Tyrosine hydroxylase (TH) | 1:5000 | Pel-Freez | | | Rogers, AR, USA |
| Neuron-specific class III ß-tubulin (TuJ1) | 1:5000 | Covance | | | Richmond, CA, USA |
| SMAD1/5/8 | 1:1000 | Santa Cruz Biotechnology | | | Santa Cruz, CA, USA |
| Monoclonal antibody (Mouse) | | | | | |
| Nestin | 1:500 | BD Biosciences | | | Franklin Lakes, NJ, USA |
| TH | 1:2000 | Sigma-Aldrich | | | St. Louis, MO, USA |
| TuJ1 | 1:2000 | Covance | | | Richmond, CA, USA |
| Ki67 | 1:100 | Novocastra | | | Newcastle, UK |
| β-actin | 1:5000 | Abcam | | | Cambridge, MA, USA |
| Polyclonal antibody (Goat) | | | | | |
| SMAD2/3 | 1:1000 | Santa Cruz Biotechnology | | | Santa Cruz, CA, USA |
