# Supplementary material for: Noggin Over-Expressing Mouse Embryonic Fibroblasts and MS5 Stromal Cells Enhance Directed Differentiation of Dopaminergic Neurons from Human Embryonic Stem Cells
Source: PLoS One. 2015 Sep 18;10(9):e0138460. doi: 10.1371/journal.pone.0138460 (PMC4575120; doi:10.1371/journal.pone.0138460)
Supplement: S1 Fig — (DOCX) [file pone.0138460.s003.docx]

**Supplementary Figure**


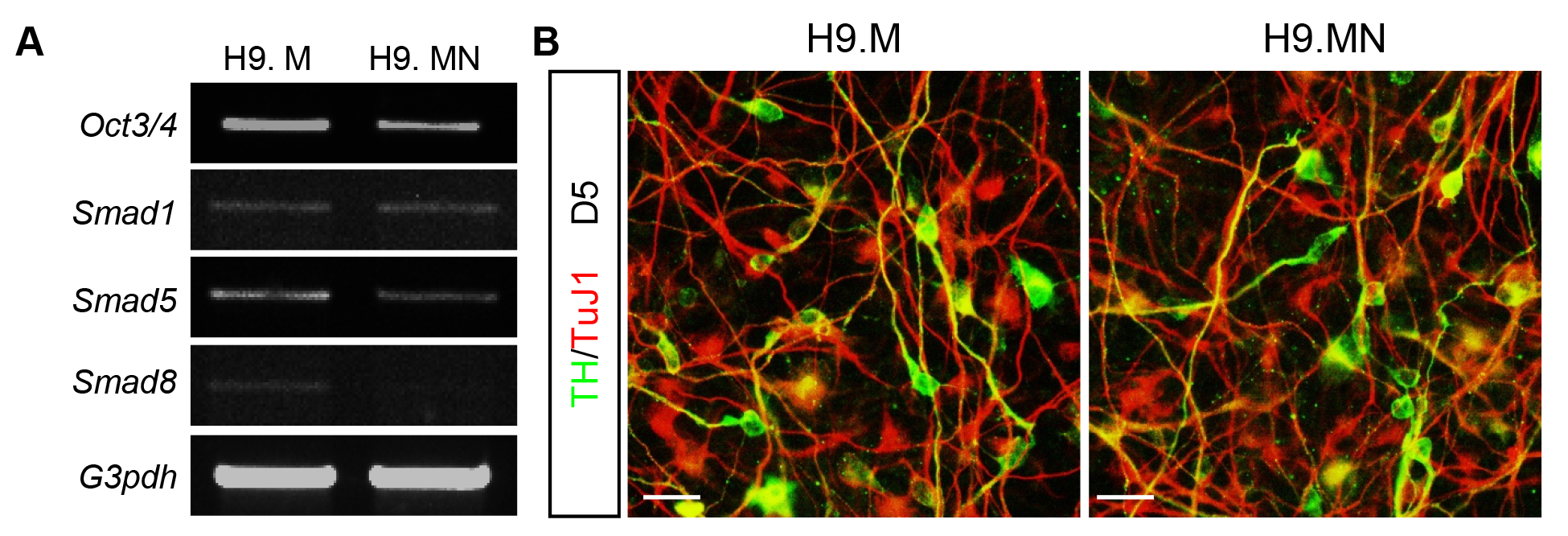


**S1 Fig. Noggin does not enhance the dopaminergic differentiation of H9 hESCs.**

To determine whether DA neuron differentiation of H9 hESCs was enhanced using noggin-overexpressing feeder cells, MS5 vs. noggin-MS5 culture systems were compared. (A) *Smad1* gene expression showed no significant difference between these two culture systems; in contrast, *Smad5, 8* gene expressions were lower in the noggin-MS5 condition than that of MS5 condition. (B) However after dopaminergic neuronal differentiation, TH expression as assessed by immunofluorescence staining showed no significant differences between the two culture systems. Scale bar = 20 μm.
